# Eye-movement signatures of syntactic development: real-time mapping of passive sentence comprehension in children aged 6–10

**DOI:** 10.3389/fnbeh.2026.1775487

**Published:** 2026-05-08

**Authors:** Olga V. Kruchinina, Daria W. Lundina, Tatyana A. Balabanova, Nataliya V. Makurina, Elizaveta I. Galperina

**Affiliations:** Research Group of Developmental Psychophysiology, Sechenov Institute of Evolutionary Physiology and Biochemistry RAS, St. Petersburg, Russia

**Keywords:** age differences, cognitive development, eye-tracking, oculomotor activity, passive voice, Russian, sentence-picture matching, syntactic revision

## Abstract

**Introduction:**

The ability to understand complex sentences, such as passives, improves during middle childhood. However, it remains unknown when children transition from a “wait-and-listen” strategy to incremental, word-by-word revision of interpretive hypotheses, and whether behavioral accuracy reflects mature online processing.

**Methods:**

Using eye-tracking in a sentence-picture matching task, we examined how Russian-speaking children aged 6–7 years (*n* = 16), 8–10 years (*n* = 16), and adults (*n* = 35) process four sentence types (active/passive, direct/reversed word order). Semantic cues were minimized, forcing reliance on morphosyntactic markers. Linear mixed models treated age as a continuous variable to capture fine-grained trajectories.

**Results:**

Accuracy improved sharply between ages 7 and 8, with 8–10-year-olds performing at adult levels. However, oculomotor patterns revealed a clear dissociation: at the critical second word in passive direct sentences—where the participle signals thematic role revision—adults and 8–10-year-olds showed a distinct signature (decreased fixation time, increased gaze returns), indicating rapid incremental revision. This signature was absent in 6–7-year-olds, who delayed engagement until the third word. Linear mixed models confirmed that age-related increases in fixation duration were specifically tied to this revision point, extending previous ERP findings that localized revision effects only to the third word.

**Discussion:**

Adult-like behavioral accuracy by age 8–10 masks continued immaturity of incremental revision mechanisms. Eye-tracking captures this dissociation, positioning it as a sensitive marker of syntactic development and revealing that the ability to use morphosyntactic cues for real-time revision continues to develop beyond middle childhood.

## 1 Introduction

### 1.1 Semantic and syntactic development in childhood

Real-time language comprehension relies on both semantic and syntactic analysis. Children begin to understand spoken language by analyzing the semantics of words in context, much earlier than they rely on syntactic markers (Strotseva-Feinschmidt et al., 2019). A critical aspect of this developmental trajectory is the dynamic weighting of semantic and morphosyntactic cues during online comprehension. While adult language processing rapidly integrates both sources of information, young children initially rely heavily on semantic plausibility. Behavioral and neuroimaging studies demonstrate that 5-year-olds prioritize semantic information over syntactic cues when these conflict, with neural activation for syntax in the left fronto-temporal network increasing only later with development (Wu et al., 2016). Cross-linguistically, children below age seven struggle to use morphological case marking independently of word order, often defaulting to canonical agent-action-patient schema even when case markers indicate otherwise (Dittmar et al., 2008). This difficulty stands in contrast to children’s early-emerging ability to use semantic information predictively: by age three, children can anticipate upcoming nouns based on verb semantics (Mani and Huettig, 2012; Borovsky and Creel, 2014). The developmental lag between semantic prediction and morphosyntactic revision suggests that these processes rely on partially distinct mechanisms, with the latter placing greater demands on cognitive control (Novick et al., 2005). German-speaking children, for instance, fail to use case marking independently of word order until age six or seven, despite being able to use lexico-semantic information predictively at age four (Dittmar et al., 2008; Schipke et al., 2012). Similar developmental patterns are observed in the comprehension of object relative clauses, where children initially misinterpret the first noun as the agent and must revise their initial hypothesis (Edeleva, 2023). This suggests a developmental asymmetry: sensitivity to semantic cues emerges early and guides initial interpretations, while the consistent and rapid integration of morphosyntactic markers—particularly when they require revision of a semantically plausible but syntactically incorrect hypothesis—continues to mature throughout middle childhood (Skeide et al., 2014). The transition from reliance on semantic plausibility to the consistent use of morphosyntactic cues when assigning thematic roles is a key step in grammatical development (Dittmar et al., 2008; Unsworth, 2016), supported by both linguistic experience and the maturation of attentional and executive systems (Friederici et al., 2011; Akhutina et al., 2019).

### 1.2 The Russian language as a test case

The Russian language provides an ideal context for studying this transition because morphological case marking—rather than fixed word order—determines the agent and patient in an event (Sekerina, 2003; Kazanina, 2011; Bailyn, 2011; Akhutina et al., 2017, 2019). Consider the sentences “The panda painted the zebra” and “The zebra painted the panda.” In English, word order alone disambiguates who did what to whom; the subject comes first (subject-verb-object, SVO). In Russian, however, both orders are possible, and thematic roles are signaled by case endings: “*Panda (noun.NOM) pokrasila/painted (verb) zebr****u*** *(noun.ACC)*” (SVO) means “The panda painted the zebra,” while “*Zebr****u*** *(noun.ACC) pokrasila/painted (verb) panda (noun.NOM)*” (OVS) means the same—“The panda painted the zebra”—despite the reversed word order. To accurately understand OVS sentences (e.g., the meaning of inverses or passives), children must detect subtle morphological markers and integrate them rapidly during auditory processing. Critically, the present study’s design eliminates semantic bias entirely: by using animate, size-matched agents and patients, we remove any plausibility cues, forcing participants to abandon heuristic strategies and rely exclusively on morphosyntactic markers. This allows us to isolate the precise developmental window during which children shift from semantics-dominated processing to the adult-like integration of grammatical cues for thematic role assignment.

### 1.3 Incremental processing and revision of interpretive hypotheses

In adults, sentence comprehension unfolds incrementally: listeners form and revise hypotheses about thematic roles as each word is heard (Altmann and Kamide, 2009). Evidence from parsing revision studies indicates that 5–6-year-olds often fail to revise initial misinterpretations online, even when they possess the grammatical knowledge to do so (Trueswell et al., 1999; Choi and Trueswell, 2010). By age 7–8, children begin to show adult-like revision patterns, but the efficiency of this process continues to improve throughout middle childhood (Lassotta et al., 2024; Omaki and Lidz, 2014). When input contradicts the standard SVO pattern—as in active object-initialized sentences (AR) or passive direct voice (PD) sentences—reanalysis and revision are triggered, reflected in both the characteristics of the evoked brain response (e.g., the P600 component indexing syntactic reanalysis; Hahne et al., 2004; Friederici et al., 2011) and behavioral responses, such as increased processing times and characteristic eye movement patterns such as regressions and prolonged fixations (Spivey-Knowlton et al., 1993; MacDonald et al., 1994; Traxler et al., 2002). In passive constructions in Russian, the critical point of correction is the second word (participle), for example “*Zebra (noun.NOM) pokra****šena*** *(participle) pand****oj*** *(noun.INS)*” (“The zebra is painted by the panda”), whose morphology indicates thematic role inversion. This structure thus functions as a natural “garden path” (Ferreira and Clifton, 1986), requiring the listener to revise an initial interpretive hypothesis. The present study asks whether children exhibit a similar revision pattern when the second word contradicts the SVO expectation, and at what age this ability emerges. However, an important nuance is introduced by the “good enough” parsing framework (Ferreira and Patson, 2007), which suggests that comprehenders do not always compute a full, detailed syntactic representation. Instead, they may generate a shallow or heuristic-based interpretation that is “good enough” for the task at hand, particularly under conditions of limited cognitive resources or time pressure. This framework is especially relevant for developmental research: children’s adult-like behavioral accuracy on complex sentences may sometimes reflect “good enough” interpretations that suffice for the sentence-picture matching task, even if their underlying syntactic representations remain incomplete or non-adult-like.

The Visual World Paradigm (VWP; Tanenhaus et al., 1995) has been instrumental in revealing how visual attention is dynamically coordinated with spoken language comprehension (Huettig et al., 2011; Sekerina et al., 2016). By tracking eye movements time-locked to each word, researchers can infer when and where listeners direct attention, providing a continuous, word-by-word window into the unfolding process of hypothesis formation and revision. Eye-tracking studies of German children have shown that children and adults display qualitatively similar processing patterns, although the canonical SVO interpretation persists longer in children (Adani and Fritzsche, 2015). Children, like adults, can anticipate upcoming information (Havron et al., 2020), using semantic knowledge to actively predict words rather than passively perceiving them (Borovsky and Creel, 2014; Mani and Huettig, 2014; Pickering and Gambi, 2018). However, adults uniquely use morphological cues to initiate reanalysis and revise initial interpretations online (Adani and Fritzsche, 2015).

### 1.4 From EEG to eye-tracking: complementary insights

EEG studies have established that neural sensitivity to morphosyntactic cues emerges early but undergoes prolonged maturation (Friederici et al., 2011; Hahne et al., 2004). In Russian-speaking children, ERP responses to passive voice inflections are detectable by age 4–6, but reliable differences between active and passive sentences appear only at the third word, where thematic roles are disambiguated (Kruchinina et al., 2022a,b). However, ERPs are limited in their ability to reveal how children use this information incrementally to guide attention, form hypotheses, or revise interpretations in real time. While ERPs index neural computations averaged across trials and time-locked to discrete events, they cannot capture the continuous, word-by-word dynamics of attention allocation or the strategic deployment of gaze to test interpretive hypotheses during a single trial. Eye-tracking addresses this gap by providing a continuous measure of overt attention during sentence comprehension, revealing not merely that the brain distinguishes constructions, but how listeners deploy attention to test and revise hypotheses about “who did what to whom” (Huettig et al., 2011; Wendt et al., 2014). By extending our previous ERP work to oculomotor dynamics, the present study aims to characterize the developmental progression of processing strategies—from passive end-of-sentence decision-making to active, incremental hypothesis testing.

The choice of oculomotor parameters in this study is grounded in their established sensitivity to specific cognitive processes. Fixation duration and fixation count serve as reliable indices of attentional engagement and processing difficulty: longer and more frequent fixations on a target picture reflect the time needed to integrate linguistic input with visual referents during incremental interpretation (Rayner, 1998, 2009; Demberg and Keller, 2008). Saccade frequency and gaze returns to an area of interest (AOI) are particularly informative for tracking reanalysis and hypothesis revision—core components of syntactic processing when initial parsing strategies fail. Regressive saccades have been consistently linked to syntactic reanalysis in reading (Rayner, 1998; Traxler et al., 2002), and analogous patterns emerge in the VWP (Huettig et al., 2011). Crucially, interpreting age-related differences in these metrics requires situating them within the broader development of oculomotor control. Luna and colleagues (Luna et al., 2008) demonstrated that voluntary, endogenously-driven eye movements follow a protracted maturational course, reaching adult levels only in adolescence. In the VWP, increased fixations can reflect active hypothesis testing and reanalysis rather than processing difficulty *per se* (Helo et al., 2014; Teruya, 2019; Wendt et al., 2014). Our specific parameter set—first fixation duration, total fixation time, fixation count, gaze returns, and saccade count—was selected following standardized methodological frameworks to ensure reliability and cross-study comparability (Komogortsev et al., 2010).

### 1.5 Research gap

Despite considerable progress in understanding children’s grammatical development, the real-time mechanisms underlying how syntax is processed and integrated with visual information remain insufficiently described. Critically, we do not know at what age children begin to process sentences incrementally—forming and revising hypotheses as each word unfolds—rather than adopting a “wait-and-listen” strategy that delays decision-making until the end of the sentence. Furthermore, it is unclear whether improvements in behavioral accuracy during middle childhood (e.g., by age 8–10) are underpinned by adult-like online processing strategies, or whether overt performance masks continued immaturity in the dynamic allocation of attention. This dissociation—between what children know (accuracy) and how they process (real-time attention)—can only be addressed with temporally sensitive measures such as eye-tracking. Very few studies examine eye movements word-by-word during the unfolding of auditory sentences, leaving open the question of when children extract and apply morphosyntactic information, and how they progressively construct and revise thematic role hypotheses (Sekerina et al., 2016; Huettig et al., 2011).

### 1.6 The present study: aims and research questions

A preliminary analysis of a subset of the current sample (*n* = 20, 10 children and 10 adults) was previously published in Russian (Kruchinina et al., 2024). That study reported general age differences in oculomotor behavior during passive sentence comprehension, with children exhibiting longer fixations on distractor pictures than adults. The present study substantially extends this work in several critical ways: (i) we tripled the sample size (*n* = 67), allowing for more robust statistical modeling; (ii) we divided children into two developmental groups (6–7 and 8–10 years) and also treated age as a continuous variable in linear mixed models; (iii) we analyzed eye movements word-by-word across four grammatical types (active/passive, direct/reversed word order); and (iv) we tested specific hypotheses about the role of morphosyntactic cues (second word) in triggering revision of initial thematic role assignments.

The present study investigates how Russian-speaking children aged 6–10 years process grammatical constructions of varying complexity in a sentence-picture matching task. Using eye-tracking, we examine how eye-movement metrics reflect children’s ability to extract and use morphosyntactic cues to assign thematic roles, and we determine when children’s oculomotor behavior begins to resemble that of adults. Our research questions are:

Developmental trajectory of revision: At what age do children transition from a “wait-and-listen” strategy (decision after the full sentence) to an incremental, word-by-word strategy involving active hypothesis revision? Does this transition occur uniformly across different grammatical types?
Role of the second word: How do children use the second word (verb/participle) to revise an initial SVO hypothesis in passive and reverse sentences? Do oculomotor measures (fixation time, fixation count, saccades, gaze returns) reveal sensitivity to these revision demands earlier than behavioral accuracy alone?
Differential processing of grammatical types: Are passive voice sentences with direct word order (PD) processed differently from other types (AD, AR, PR), and how does this processing signature change with age?
Dissociation between accuracy and online processing: Do children aged 8–10, who show adult-like behavioral accuracy, nevertheless exhibit immature oculomotor patterns during online processing? If so, what does this dissociation reveal about the continued maturation of syntactic processing mechanisms?

### 1.7 Hypotheses

We hypothesize that:

*H1* (Reanalysis Hypothesis): The second word serves as a critical trigger for syntactic revision. In adults and older children (8–10 years), processing of PD and AR sentences will elicit increased fixation time and saccade frequency on the target picture specifically at the second word, reflecting active hypothesis testing and reanalysis. In younger children (6–7 years), these oculomotor indices of revision will be absent or attenuated.

*H2* (Developmental Asynchrony Hypothesis): There will be a dissociation between behavioral and online measures. While accuracy for complex sentences will reach adult levels by age 8–10, oculomotor patterns in this age group will continue to differ from adults, indicating that the efficiency of incremental revision continues to develop even after overt performance has matured.

*H3* (Word-by-Word Incremental Processing Hypothesis): Mature processing is characterized by a progressive increase in visual engagement with the target picture across word positions (1 → 2 → 3), reflecting the gradual accumulation and verification of evidence for thematic role assignment. Adults and older children will show this pattern, while younger children will show a flatter trajectory, with significant engagement only at the third word or after sentence offset.

By integrating these hypotheses with fine-grained oculomotor measures, this study aims to characterize not merely when children understand complex syntax, but how they deploy attention to test and revise interpretive hypotheses in real time—and to establish eye movements as sensitive markers of grammatical maturation in childhood.

## 2 Materials and methods

### 2.1 Participants

Data collection was conducted in two stages. In the first stage, 20 participants (10 children, 10 adults) were recruited for a preliminary study (reported in Kruchinina et al., 2024, in Russian). After confirming that the paradigm was functioning correctly and no modifications were needed, we continued data collection to reach the final sample of 67. Thus, the initial 20 participants are included in the final sample. The study involved native monolingual Russian speakers, children (*n* = 36) and adults (*n* = 35). Four children were excluded from the initial sample for technical reasons, resulting in a final sample of 32 children (aged 6 to 7 years, average age 6.8 ± 0.6 years, *n* = 16, 7 females; aged 8 to 10 years, average age 9.4 ± 1.1 years, *n* = 16, 9 females) and a control group of 35 adults (aged 18 to 46 years, average age 23.1 ± 1.7 years, 31 females). None of the participants had neurological or hearing disorders, and all had normal or corrected-to-normal vision. The information was taken from the medical history based on individual medical records. To assess speech development of children, the Russian Phonological Battery for the Assessment of Language Development (“Zarya”) for children aged 7–10 years (Dorofeeva et al., 2020) and the RuCLAB (Russian Child Language Assessment Battery, “Korablik”) for children aged 6 (Gomozova et al., 2021) were used. All children demonstrated speech development in line with age norms (Supplementary Material 1).

### 2.2 Stimuli

#### 2.2.1 Linguistic stimuli

The protocol for the sentence-picture matching task was adapted for an eye-tracking study from the original program “Gramconstructor” (Certificate of State Registration No. 2020616013, St. Petersburg, Russia). Subjects had to select one picture from a pair that corresponded to the sentence they heard and then press the right or left response button, depending on whether the target picture was on the right or left. The test included four grammatical types of three-word sentences (*n* = 24 for each type, totaling 96): active voice with direct word order (AD), active voice with reversed word order (AR), passive voice with direct word order (PD) and passive voice with reversed word order (PR). An example of the linguistic stimuli is presented in Table 1 and Supplementary Material 2. Pairs of animate nouns were selected as the subject and object of the action, with each pair sharing the same grammatical gender, number of syllables (two or three), and frequency (no less than 10 items per million (ipm), according to the corpus of the Russian language (Russian National Corpus, 2018) and number (only single). Sentences were constructed so that each noun acted as both the subject and the object of the action (e.g., The grandchild hugged the grandfather. The grandfather hugged the grandchild).

**TABLE 1:** An example of a thematic relationship expressed through four grammatical types in the Russian language.

Sentence grammatical type (Russ.)	Word order (Russ.)	First word (noun 1)	Second word (verb/participle)	Third word (noun 2)
Active voice	“The panda painted the zebra.”			
	Direct word order (AD)	*/Panda* (noun.NOM) panda	*pokras****ila*** (verb) painted	*zebr****u***./ (noun.ACC) zebra
	Syntactic construction	S	V	O
	Reversed word order (AR)	/*Zebr****u*** (noun.ACC) zebra	*pokras****ila*** (verb) painted	*panda.*/ (noun.NOM) panda
	Syntactic construction	O	V	S
Passive voice	“The zebra is painted by the panda.”			
	Direct word order (PD)	/*Zebra* (noun.NOM) zebra	*pokra****šena*** (participle) is painted	*pand****oj***./ (noun.INS) panda
	Syntactic construction	O	V	S
	Reversed word order (PR)	/*Pand****oj*** (noun.INS) panda	*pokra****šena*** (participle) is painted	*zebra.*/ (noun.NOM) zebra
	Syntactic construction	S	V	O

Grammatical markers are highlighted in bold.

#### 2.2.2 Audio stimuli

All sentences were recorded in a sound-insulated anechoic chamber to ensure optimal acoustic conditions. A female voice was selected for the recordings. After recording, the duration of each stimulus (word) was time-aligned to 1300 ms, including pre- and post-stimulus intervals, i.e., duration of each sentence is 3900 ms, regardless of the conditions.

#### 2.2.3 Visual stimuli

**FIGURE 1:**
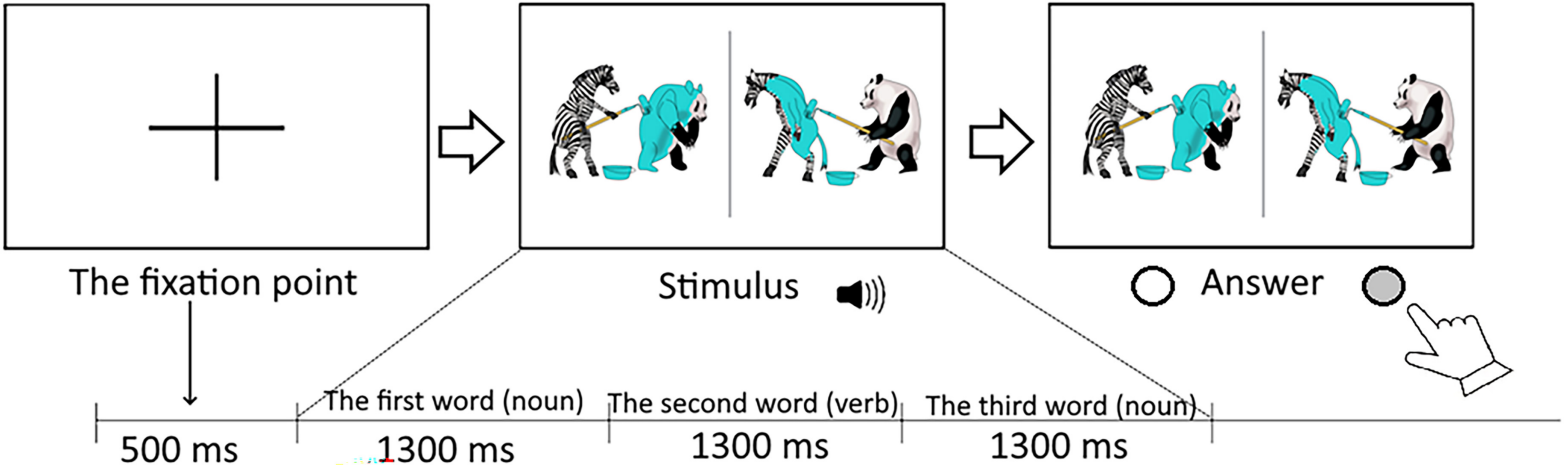
An experimental design. A sentence is presented through the headphones: The panda painted the zebra (AD).

Each sentence corresponded to one of a pair of colored pictures (*n* = 12 pairs) depicting the subject and object of the action (humans or animals). The pictures were paired and symmetrical plot, meaning that the direction of action is symmetrical on each pair of pictures. All pictures adhered to the following principles: (1) the object and the subject were clearly identifiable, as well as action connecting them; (2) the pictures contained no extraneous details that were meaningless for comprehension; (3) colors in the pictures were balanced, with no more than four colors used in each; (4) the subject and object of the action occupied comparable areas (with a difference of no more than 25%); (5) the elements of the picture did not intersect. An example of the visual stimuli is shown in Figure 1.

#### 2.2.4 Validation

In order to exclude pictures and audio stimuli that could be ambiguously interpreted by the subjects from the study, the stimuli were validated before the experiment. Details of the validation procedure are described in Kruchinina et al. (2022b,2024).

### 2.3 Design and procedure

The study was conducted in a laboratory setting. Children were seated in a car seat that was securely fastened to a chair in front of the monitor. All subjects performed a sentence-picture matching task (Figure 1). On each trial, two pictures (target and distractor) appeared simultaneously on the screen, centered in the left and right halves of the display, and an audio recording of the sentence was played. During the task, oculomotor activity was recorded using a stationary eye-tracker (GP3 Desktop, 60 Hz, Canada) with a reported accuracy of 0.5–1°. The eye tracker performed binocular tracking, and eye movements were classified based on a saccade velocity threshold of 150°/s.

The distance between the eye tracker and the monitor was 31 cm. The monitor (Lenovo IdeaPad 330, 15.6-inch / 40 cm diagonal) was positioned at a 35° angle relative to the participant’s eyes and the table surface. The angular dimensions of the stimuli were 11.7° horizontally and 20.8° vertically. Room illumination ranged from 200 to 300 Lx, with incandescent lamps used and direct sunlight excluded. Audio-recorded sentences were presented via JBL Tune 500 Black over-ear headphones.

To prevent fatigue and maintain optimal performance, the 96 stimulus sentences were divided into four presentation blocks, with short breaks between them. Each block lasted approximately 4 min, and the entire study session did not exceed 25 min. Block order was randomized across participants. Within each block, stimuli were balanced for laterality of the correct answer (right/left side of the monitor) and grammatical type. Each block contained six sentences of each grammatical type (AD, AR, PD, PR), and each picture pair appeared twice.

Before each block, a 9-point calibration was performed. Calibration was considered successful when accuracy and precision were both below 2°.

The trial structure and timeline of the experiment is depicted in Figure 1.

### 2.4 Data analysis

The analysis of oculomotor reactions was conducted using the Neurobureau software package (Komogortsev et al., 2010, Skuratova et al., 2022), specifically the Gazeanalyzer section (Russia, 2019). The target and distractor pictures were identified as separate areas of interest (AOIs) for each stimulus, with each AOI covering 50% of the monitor. The target picture appeared in the left and right halves of the screen, with its position counterbalanced across different grammatical types. Oculomotor activity was recorded while participants listened to the sentence. Oculomotor activity data for each word in the sentence, specifically for the ROI corresponding to the presented sentence, taking into account the correctness of the answer, were collected for further analysis. Artifactual data (eye tracker gaze loss) were excluded from the dataset: 7.6% in adults and 16.5% in children. The characteristics of oculomotor activity that were analyzed included:

- the duration of the first fixation (*first fix.*, [sec]);
- the duration of all fixations (*fix. time*, [sec]);
- the total number of fixations (*all fix.*, [*n*]);
- the number of gazes returns to the AOI (*returns*, [*n*]);
- the number of saccades (*saccade count*, [*n*]).

For each subject, the above-mentioned parameters of oculomotor activity were averaged separately in each of the four types of sentences for the 1st, 2nd, and 3rd words.

### 2.5 Statistical analysis

Data collection proceeded in two phases with no modifications to the experimental design, and the initial 20 participants were included in the final sample of 67 (32 children aged 6–10 years and 35 adults). A *post hoc* power analysis using G*Power (Faul et al., 2007) was conducted to verify the adequacy of the final sample for the mixed-design ANOVA (between-subjects factor: 3 age groups; within-subjects factors: 4 sentence types × 3 word positions). With a total sample size of *n* = 67, an α-level of 0.05, a medium effect size (*f* = 0.25), and an assumed correlation among repeated measures of *r* = 0.5, the achieved power exceeded 0.99. This confirms that the sample provided sufficient statistical power to detect the effects of interest in the planned analyses. To further address potential concerns regarding the inclusion of the initial 20 participants in the final sample, we conducted an additional sensitivity analysis excluding these participants (*n* = 47). This analysis yielded an achieved power of 0.99, confirming the robustness of our sample size even under a more conservative scenario.

A Shapiro–Wilk test was used to assess the normality of data distribution. As the distribution was non-normal, the equality of variances was evaluated using Mauchly’s test of sphericity criterion to identify within–group differences, since the null hypothesis was rejected, the Gringhouse-Geisser amendment was adopted.

A general linear model (GLM) mixed-design ANOVA was employed to compare behavioral data and characteristics of oculomotor activity in each age group (6–7 y.o., 8–10 y.o. and adults) during the task. The behavioral data (percentage of correct responses) was analyzed using two factors: between-subject (Age group, 3 levels) and within-subject (Type of sentences, 4 levels), along with the interaction between these two factors. To examine the relationship between age differences and characteristics of oculomotor activity, a mixed-design GLM was conducted. This model included age group as a between-subject factor with three levels, and two within-subject factors: type of sentences with four levels and word position with three levels. Sidak’s correction was applied (*p* < 0.01). Data are presented as mean ± standard deviation. The statistical analysis was conducted using IBM SPSS software (version 26).

In addition to the group analysis, we conducted an additional linear mixed model (LMM) analysis using only the child data (*n* = 32) to treat age as a continuous variable. This approach allowed us to model the modulating effect of age as a continuous covariate (centered at 8 years) on the oculomotor function parameters. Separate LMM models were constructed for each dependent variable (fixation time, total number of fixations, number of saccades). Fixed effects included age, sentence type (4 levels), word position (3 levels), and all their interactions. Random effects included a random intercept for participants to account for individual variability. Significant three-way interactions were decomposed, and Sidak’s correction was applied for multiple comparisons in the *post hoc* analysis of the effect of age in each specific condition (sentence type × word position). We modeled the data with mixed-effects linear regressions in the R software^1^ using the lmer function from the lme4 package (Bates et al., 2015). To obtain p values from the t values given by the model, we used the lmerTest package (Kuznetsova et al., 2017). Random intercepts by participant and by item were included in the models. Emmeans package (Lenth and Piaskowski, 2025) was used for pairwise comparisons. *P*-values were adjusted using the Sidak method.

## 3 Results

### 3.1 Behavioral data

The sentence picture-matching test was assessed based on the number of correct answers; reaction time was not measured. The percentage of correct answers indicated age-related specificity in the acquisition of passive voice and reversed word order. A mixed-design GLM, incorporating the within-subject factor “Sentence Type” (4 levels: AD, AR, PD, PR) and the between-subject factor “Age” (3 levels: 6–7 y.o., 8–10 y.o., adults), indicated a significant effect of participants’ age on the percentage of correct answers [*F*(2, 64) = 17.96, *p* < 0.0001, η^2^ = 0.29].

**FIGURE 2:**
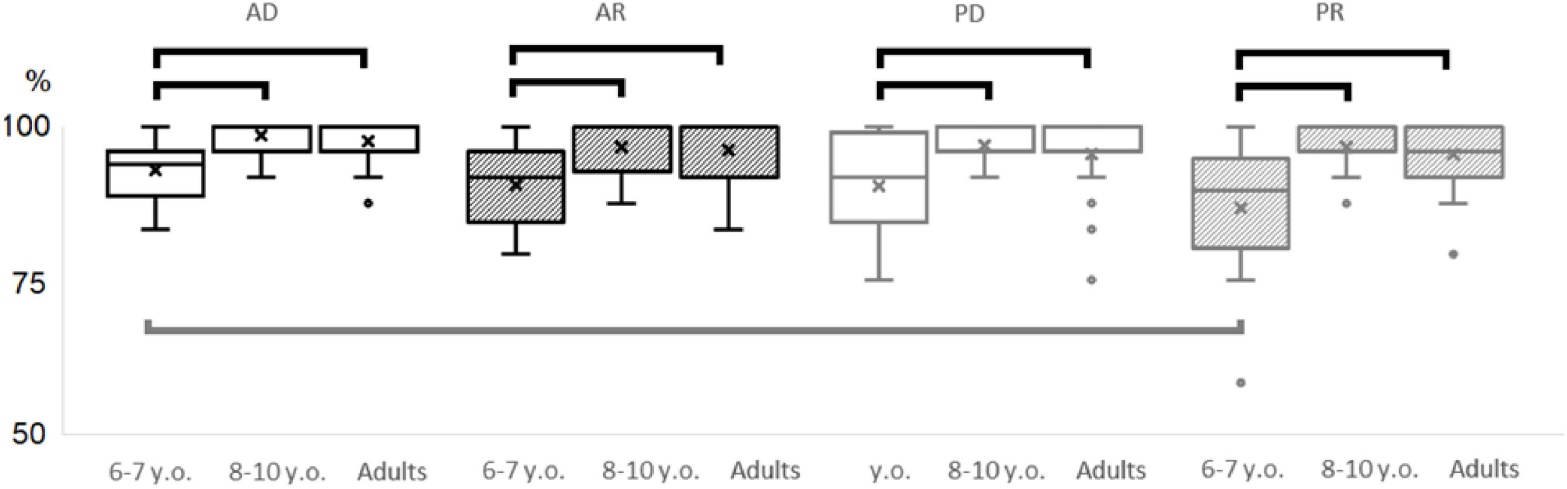
The percentage of correct answers in the sentence picture-matching test. AD, active voice with direct word order; AR, active voice with reversed word order; PD, passive voice with direct word order; PR, passive voice with reversed word order. Black line—age group differences in percentage of correct answers in each grammatical type (*p* = 0.001). Grey line—the difference in percentage of correct answers AD vs. PR in 6–7 years old children (*p* = 0.001).

Overall, in all grammatical types, children aged 6–7 had a lower percentage of correct answers compared to children aged 8–10 and adults (Figure 2). Differences in accuracy between the sentence types were observed only among children aged 6–7 years. Children exhibited a significantly lower percentage of correct answers for PR sentences compared to AD (*p* = 0.001). Therefore, a significant improvement in comprehension of complex syntax occurs between the ages of 7 and 8.

### 3.2 Oculomotor behavior

All data described relate to oculomotor behavior in the target (matching) picture. A mixed-design GLM with Sentence Type (AD, AR, PD, PR) × Word Position (1st, 2nd, 3rd word) as within-subject factors and Age Group (6–7, 8–10 years old children and adults) as a between-subject factor revealed a significant effect of “Word Position” and “Sentence Type” on all examined oculomotor activity parameters, with the exception of *returns* for “Sentence Type” (Table 2). The interaction of the factors “Type Sentence * Word Position * Age group” was only evident in *fix. time* and *saccade count* (Table 2).

**TABLE 2:** The influence of within-subject factors (sentence type and word position) and their interaction with between-subject factors (age group) on oculomotor parameters in a mixed GLM design.

Oculomotor parameters	Factor	*F*	df	Error degrees of freedom	*p*	η ^2^
First fixation	**Sentence type**	**2.91**	**3**	**192**	**0.04**	**0.04**
	**Word position**	**68.30**	**2**	**128**	**0.00**	**0.51**
	Sentence type ^×^ Word position^×^ Age group	1.15	12	384	0.32	0.04
All fixation	**Sentence type**	**9.35**	**3**	**192**	**0.00**	**0.13**
	**Word position**	**19.48**	**2**	**128**	**0.00**	**0.23**
	Sentence type ^×^ Word position^×^ Age group	1.51	12	384	0.12	0.05
Fixation time	**Sentence type**	**7.01**	**3**	**192**	**0.00**	**0.1**
	**Word position**	**271.49**	**2**	**128**	**0.00**	**0.8**
	**Sentence type ^×^** **Word position^×^** **Age group**	**3.23**	**12**	**384**	**0.01**	**0.1**
Returns	Sentence type	1.23	3	192	0.3	0.02
	**Word position**	**30.87**	**2**	**128**	**0.00**	**0.33**
	Sentence type ^×^ Word position^×^ Age group	1.57	12	384	0.09	0.05
Saccade count	**Sentence type**	**11.56**	**3**	**192**	**0.00**	**0.15**
	**Word position**	**5.58**	**2**	**128**	**0.01**	**0.1**
	**Sentence type ^×^** **Word position^×^** **Age group**	**2.39**	**12**	**384**	**0.01**	**0.07**

Significant differences are indicated in bold.

#### 3.2.1 Age

The mixed-design GLM analysis revealed a significant main effect of the between-subject factor (age group) on fixation parameters, including *all fix*. [*F*(2, 64) = 5.97, *p* < 0.01, η^2^ = 0.16], *fix. time* [*F*(2, 64) = 18.14, *p* < 0.001, η^2^ = 0.34] and *returns* [*F*(2, 64) = 6.04, *p* < 0.01, η^2^ = 0.16].

**FIGURE 3:**
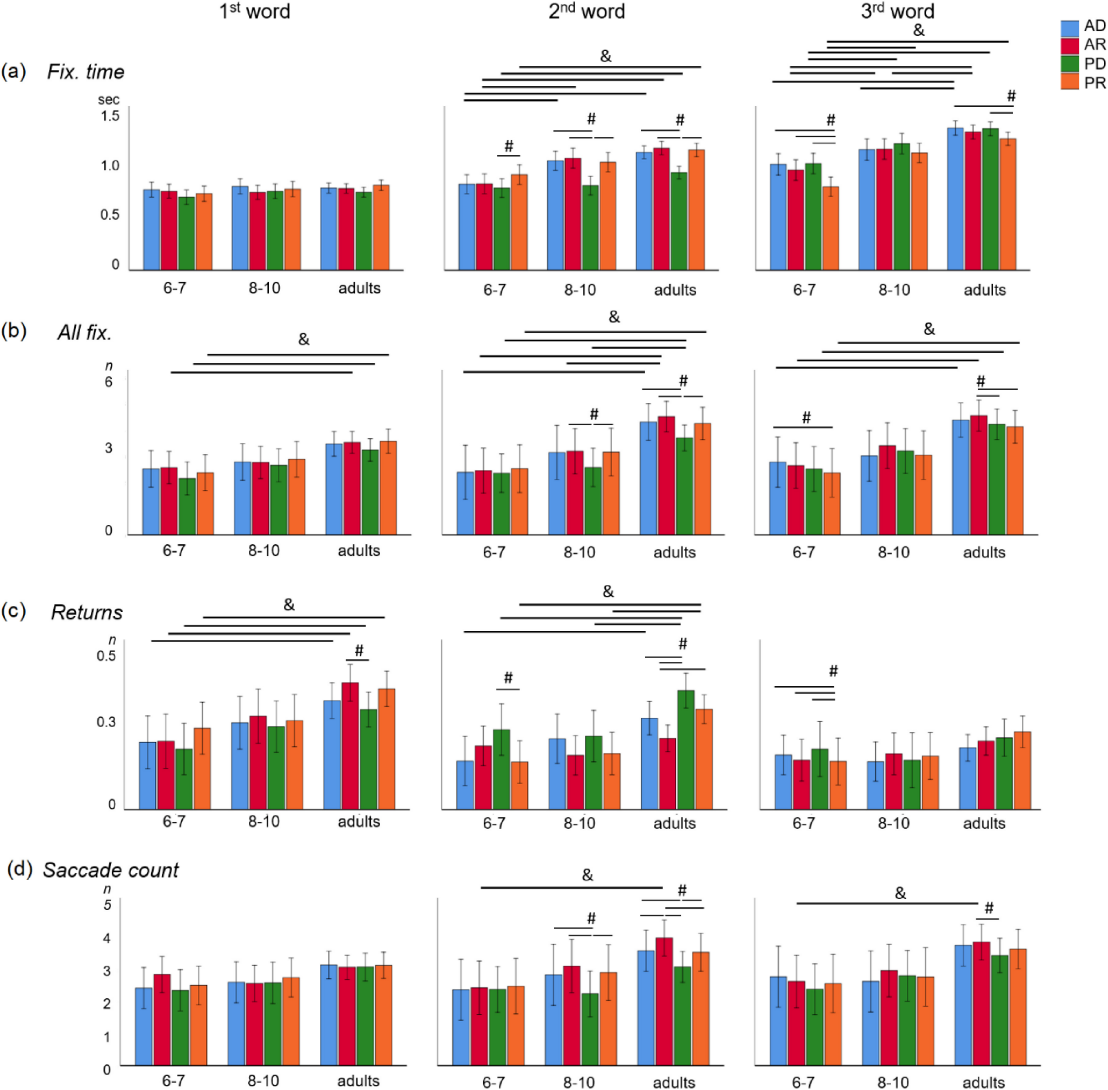
Oculomotor activity on the target picture in children (6–7 years old: *n* = 16; 8–10 years old: *n* = 16) and adults (*n* = 35) during sentence comprehension across different grammatical types. Data presented only for target picture AOI. **(a)** Fixation times, **(b)** all fixations, **(c)** returns, **(d)** saccades count. The grammatical types: AD, active voice with direct word order (blue); AR, active voice with reversed word order (red); PD, passive voice with direct word order (green); and PR, passive voice with reversed word order (orange). & – age differences (*p* < 0.05). # – between grammatical types differences (*p* < 0.001). Error bars represent SEM.

The *post hoc* Sidak’s correction revealed differences in oculomotor activity between children aged 6–7 years and adults during the selection of the target picture. Additional models testing differences in eye movements between age groups, adjusted for multiple comparisons, are provided in Supplementary Materials 3, 4. Figure 3 illustrates that when listening to the 2nd and 3rd words, adults had significantly higher fixation times on the target picture than 6–7-year-old children (*p* < 0.0001) for all sentence types, and for the third word for active-voice sentences, they were higher than for 8–10-year-old children (*p* < 0.02). A greater number of all fixations were found in adults compared to 6–7-year-old children for each word, and between 8 and 10-year-old children and adults for the second word. The number of returns to the target image was higher in adults compared to 6–7-year-old children for the first and second words, and compared to 8–10-year-old children for the second word. This suggests that, even when listening to the 1st and 2nd words in a sentence, adults already have a hypothesis about thematic roles, which they test by analyzing the target picture and do not return to analyzing the distractor. Children aged 8–10 years begin to use a similar strategy for some types of sentences (for example, AD), and children aged 6–7 years need to listen to the entire sentence before starting to analyze and make a decision.

Age differences between younger and older children were noted only for *fixation times*: for active voice sentences, children aged 8–10 had longer fixation times when listening to the 2nd word (AD: *p* = 0.003, AR: *p* = 0.002) and on the 3rd word (AR: *p* = 0.02) compared to children aged 6–7. Together, these results suggest a qualitative shift in oculomotor strategies between 6–7 y.o. and 8–10 y.o. despite similar behavioral accuracy for 8–10 y.o. and adults.

#### 3.2.2 Grammatical type

The *post hoc* analysis using Sidak’s correction revealed differences in oculomotor activity during the comprehension of sentences with various grammatical types (Figure 3 and Supplementary Material 4). The most significant differences were observed in sentences with PD. In both adults and 8–10-year-old children, these differences are most pronounced at the second word in the sentence, where PD exhibits significantly shorter *fix. time*, *all fix.*, and fewer *saccades* on the target picture compared to other sentence types. In 6–7-year-old children, differences between PD and PR sentences begin to emerge at the second word, showing shorter *fix. time* but more *returns* to AOI.

Regarding sentences with reversed word order, adults demonstrate a higher number of returns in AR on the first word compared to PD. At the second word, there are more *saccades* and fewer *returns* compared to all other sentence types. The third word shows a higher *all fix.* and *saccades* on AR compared to both PD and PR (Figure 3). For sentences with PR, adults exhibit shorter *fix. time* on the third word compared to AD and PD sentences. In children aged 6–7, the differences between PR and other sentence types are evident through shorter *fix. time, all fix.* and *returns* during the third word.

#### 3.2.3 Word position

As sentences unfold, online processing occurs, marked by changes in oculomotor activity on the target picture (Table 2). The *first fix., all fix, fix. time* and *saccade count* consistently increase from the 1st word to the 3rd, while the number of *returns* to the AOI decreases in adults and 8–10-year-old children (Supplementary Material 4). In 6–7-year-old children, we also observe signs of online processing, reflected only in a significantly longer *first fix.* and *fix. time* on the third word compared to the first, along with a decrease in returns to the ROI to the 3rd word compared to the 1st. In 6–7-year-old children, a gradual increase in fixation time from the 1st to the 3rd word is observed in AD, AR, and PD sentences (*p* < 0.01), but not in PR sentences. In 8–10-year-old children, *fix. time* for the 1st word significantly differs from that of the 3rd word across all sentence types, although the *saccade count* did not show a difference. In adults, there are clear distinctions in oculomotor parameters among words 1, 2, and 3 (Figure 3 and Supplementary Material 4).

These findings indicate that, during online processing, the establishment of word position develops gradually with age, starting with the most common sentence structures (SVO), such as those featuring active direct speech.

#### 3.2.4 Continuous age effects within the child group

To further explore developmental trajectories across childhood, we supplemented our group analysis with linear mixed models (LMM) for eye movement parameters, treating age as a continuous variable from 6 to 10 years, centering the covariate at age 8 (Supplementary Material 5). The goal of this analysis was to identify the specific language contexts in which annual maturation occurs.

For *first fix.*, significant main effects were found for sentence type [*F*(3, 352) = 3.00, *p* = 0.030] and word position [*F*(2, 352) = 67.95, *p* < 0.001], as well as their interaction [*F*(6, 352) = 2.30, *p* = 0.034]. The interaction between word position and age approached significance (*p* = 0.064). This indicates that the pattern of word recognition during early processing stages varies depending on the sentence context and is possibly modulated by age.

**FIGURE 4:**
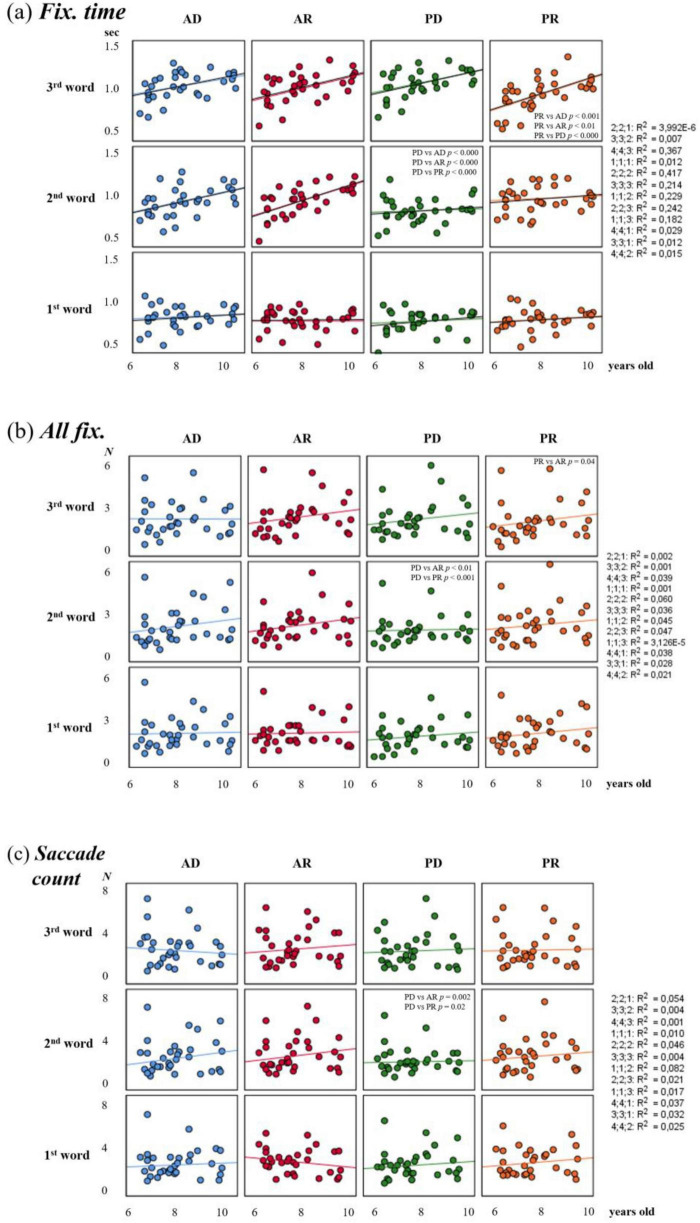
Detailed developmental trajectories for the children. Panels show age effects for the eye movement parameters interacting with sentence type, word number, and age in linear mixed models (LMMs) with age treated as a continuous variable (centered at 8 years). Each point represents an individual participant (*n* = 32 children aged 6–10 years). Regression lines illustrate the positive relationship between age and eye movement parameters. **(a)** Fixation time (in seconds), **(b)** total number of fixations (*all fix.*, n), **(c)** saccade count (n).

For gaze fixation time, the linear mixed model revealed a significant positive main effect of sentence type [*F*(3, 352) = 3.41, *p* = 0.018], word position [*F*(2, 352) = 177.87, *p* < 0.001], and age [*F*(1, 33) = 10.05, *p* = 0.003], indicating an overall increase with age. Additionally, a significant three-way interaction of sentence type × word position × age was found [*F*(6, 352) = 3.39, *p* = 0.003]. To interpret the two-way components, pairwise comparisons were conducted controlling for the covariate age using *post hoc* analysis with Sidak’s correction. They showed that when listening to the second word in PD sentences, the fixation time on the target image was significantly shorter than for all other sentence types (*p* < 0.001), whereas when listening to the third word, the shortest fixation time was characteristic of the PR type (Figure 4a and Supplementary Material 5). Comparison of word positions within each sentence type revealed that for active voice sentences (AD and AR), there was an increase in fixation duration on the target image from word to word (*p* < 0.01), while for passive voice sentences, word order specificity was revealed. For PD, there was no difference between the first and second words in a sentence (*p* = 0.086), and for PR, between the second and third (*p* = 0.97). This suggests that passive voice sentences do not exhibit the age trend that is evident for active voice sentences.

For the total number of fixations (*all fix.*), a significant main effect of sentence type [*F*(3, 352) = 5.7, *p* < 0.001] and word position [*F*(2, 352) = 11.96, *p* < 0.001] was found. However, no significant main effect of age was found. However, the three-way interaction was significant [*F*(6, 330) = 2.52, *p* = 0.02]. *Post hoc* analysis with Sidak’s correction revealed differences between PD and AR and PR on the 2nd word, as well as differences between AR and PR on the 3rd (Figure 4b and Supplementary Material 5). The number of fixations on the target image from the first to the last word significantly increased only for AR and PD sentences. Thus, there is an increase in the duration (*fix. time*), but not the number of fixations (*all fix.*), as the sentences are listened to. This very specific result highlights that the improvement in visual exploration strategies with age is not global, but is closely related to the processing of passive sentences by Russian-speaking children.

A three-way interaction was also found for the number of saccades [*F*(6, 352) = 2.98, *p* = 0.007]. *Post hoc* analysis with Sidak’s correction revealed that the number of saccades was lower when listening to the second word in PD sentences than for other sentence types (Figure 4c and Supplementary Material 5).

Thus, the differences in oculomotor activity identified, adjusted for the age covariate, in terms of fixation time, number of fixations, and saccades when listening to the second word in passive-voice sentences demonstrate the peculiarities of Russian-speaking children’s analysis of this type of grammatical construction. In other words, children aged 6–10 years do not exhibit the age-related trend of increasing fixations and saccades observed for active-voice sentences.

Taken together, these detailed LMM results confirm and extend our group-level results. They demonstrate that the observed developmental shift between 7 and 8 years is not a general maturation, but rather a targeted improvement in the ability to process specific morphosyntactic cues in complex grammatical structures such as passive voice.

## 4 Discussion

The aim of this study was to assess the efficiency of grammatical processing in children aged 6–10 years compared to adults, using eye-tracking during a sentence–picture matching task. Participants listened to sentences varying in grammatical structure (active vs. passive voice, direct vs. reversed word order) and selected the target picture. This paradigm minimized semantic cues, requiring participants to rely exclusively on syntactic and morphological markers. The key finding of this study is the discovery of a qualitative shift in online speech processing strategies occurring between the ages of 6 and 10 years (specifically between 7 and 8 years). Despite the superficial similarity in behavioral performance between children aged 8–10 years and adults—manifested in high accuracy in selecting the target picture regardless of the grammatical construction type (Figure 2)—the analysis of oculomotor activity during target image selection while listening to sentences (fixation duration, number of fixations, saccades, and regressions) revealed fundamental differences in the processes of extracting and interpreting grammatical information between these age groups (Figure 3).

### 4.1 Developmental progression of syntactic processing

The results revealed a qualitative developmental shift in grammatical comprehension between the ages of 7 and 8 years, reflected in both behavioral accuracy and oculomotor dynamics. Accuracy increased across all sentence types—active, passive, direct and reversed word order—suggesting a transition to a new level of syntactic competence (Figure 2).

Our second hypothesis (H2) addressed a potential dissociation between behavioral and online measures. We predicted that despite adult-like accuracy in 8–10-year-olds (Figure 2), their underlying oculomotor patterns would continue to differ from those of adults. The data provide clear support for this hypothesis. As shown in Figure 3, although 8–10-year-old children achieved accuracy levels comparable to adults, they exhibited significantly different fixation times, numbers of fixations, and regression patterns during second-word processing. This dissociation confirms H2 and underscores a central claim of this study: behavioral mastery of complex syntax does not imply full maturation of the incremental processing mechanisms that support real-time comprehension.

Our findings extend previous ERP studies (Kruchinina et al., 2022a), which showed that “adult-like” syntactic role assignment begins around age six but continues to mature beyond this point. While prediction during sentence comprehension emerges as early as age three (Tribushinina and Mak, 2016; van Alphen et al., 2021), its efficiency depends on grammatical complexity and linguistic experience. Early studies showed that children up to the age of seven struggle to understand syntactically complex sentences, but they comprehend simple, subject-first sentences nearly perfectly (Lindner, 2003; Hahne et al., 2004; Dittmar et al., 2008). Younger children (5–6 years) often parse passive constructions only after hearing the full sentence (Stromswold, 2006). Parsing revision studies comparing 5–6-year-olds with 7–8-year-olds indicate that significant improvements in the ability to revise syntactic assumptions and utilize syntactic cues occur around the age of 7–8 (Lassotta et al., 2024). Consistent with these findings, children aged 6–7 in our study demonstrated a sequential and slower analysis strategy, making decisions primarily after hearing the entire sentence, whereas children aged 8–10 and adults began to assign thematic roles by the 2nd word (Figure 3). Around ages 7–8, children undergo a significant shift in their processing of syntax. They transition from meaning-focused strategies, which rely on semantics and vocabulary, to more syntax-based and incremental approaches (Omaki and Lidz, 2014). This shift is likely not monolithic. Our LMM analysis, which treated age as a continuous variable, captures this development with high precision. It shows that the year-by-year increase in both fixation time and the number of fixations from ages 6 to 10 is not uniform across all sentence types (Figure 4). Instead, it is specifically tied to the processing of the second word in PD sentences. This is precisely the moment when the participle is presented, and its morphology must be exploited to overcome the initial SVO bias. This aligns with syntactic bootstrapping theory, which posits that children leverage verb morphology and argument structure to infer meaning and assign thematic roles (Gleitman, 1990; Fisher et al., 2010; Babineau et al., 2024). This finding provides compelling evidence that the ability to integrate verb morphosyntactic information to revise the initial interpretive hypothesis, a key component of adult-like syntactic processing (Spivey-Knowlton et al., 1993), is still developing during this period.

This aligns with neurophysiological evidence indicating a transition by age 9 to a qualitatively new type of speech perception based on syntactic analysis (Skeide et al., 2014; Omaki and Lidz, 2014). This development coincides with the emergence of adult-like neural markers and changes in behavioral patterns. Between ages 7 and 9, advancements in syntactic processing correspond with major morphofunctional changes in the brain. These include synaptic consolidation and pruning, increased myelination in longitudinal association pathways, and alterations in cortical thickness and folding (Friederici et al., 2011; Nie et al., 2013; Bethlehem et al., 2022; Mousley et al., 2025). Generally, this “transition” is not characterized as an instantaneous overnight change in strategy but rather as an accelerated shift in preferences and mechanisms over several years, particularly between ages 7 and 9. Full “adult” specialization is often achieved later, typically in early adolescence (Skeide et al., 2014).

This improvement coincides with the onset of formal schooling in Russia, which likely changes the child’s approach to analyzing information. Lecce and colleagues demonstrated that the development of oculomotor, attentional, and executive skills is primarily supported by educational experiences and maturation (Lecce et al., 2024). Using the school cutoff methodology, Ferreira and Morrison (1994) demonstrated that while some metalinguistic skills develop with age, others, particularly those requiring working memory (such as processing long syntactic constituents), improve specifically as a result of schooling (Ferreira and Morrison, 1994). Our task, requiring integration of morphosyntactic cues across several words, likely places similar demands on working memory and may be particularly sensitive to schooling effects. Recent research on Russian-speaking children further supports this interpretation. A study of Russian-speaking children found that the transition to adult-like mechanisms of sentence comprehension occurs toward the end of primary school (approximately 9–10 years), with complex constructions involving syntactic ambiguity (e.g., participial phrases with multiple potential attachments) showing the most protracted development (Pilipets and Staroverova, 2024). This aligns with our finding that while 8–10-year-olds achieve adult-like accuracy, their oculomotor patterns continue to differ, suggesting that the consolidation of syntactic strategies is a gradual process shaped by accumulated linguistic experience both in and out of school. The school environment provides systematic exposure to complex syntax through literacy instruction and metalinguistic reflection on grammatical rules (Gordon-Pershey, 2022). Thus, the improved accuracy and more dynamic oculomotor strategies in 8–10-year-olds likely reflect the cumulative effect of both brain maturation and structured educational input, enabling the transition from semantics-based heuristics to adult-like morphosyntactic processing.

Taken together, these detailed LMM results confirm and extend our group-level findings, demonstrating that the developmental shift observed between 7 and 8 years represents a targeted improvement in the ability to process specific morphosyntactic cues at the verb position—a critical gateway to adult-like syntactic competence.

### 4.2 Eye-movement evidence for strategy shifts

Eye-tracking data can be viewed through the lens of sentence processing strategies. The maturity of these strategies can be understood as a differentiated approach to analyzing various sentence types (the “sentence” factor) and a change in online strategies as the sentence unfolds—i.e., as clarifying information is received that allows for the unambiguous interpretation of meaning (the “word” factor). In contrast to the typical patterns observed in visual scene viewing and reading, where fixation durations generally decrease and saccade frequency declines with age due to the maturation of the oculomotor system (Luna et al., 2008; Rebrejkina and Levkovich, 2024), our spoken sentence comprehension and picture-matching task demonstrated an age-related increase in both the total number of fixations and the number of saccades on the target image as the sentence progressed. This suggests a progressive hypothesis-testing process during auditory processing (Figures 3, 4 and Supplementary Materials 4, 5). Specifically, children aged 8–10 years and adults exhibited significantly more fixations and saccades compared to younger children (6–7 years), particularly when processing the second and third words in sentences. Furthermore, older children and adults began utilizing grammatical cues, such as verb morphology, to establish subject-object relations as early as the second word, while younger children delayed these computations until the third word or even the end of the sentence. Our third hypothesis (H3) predicted that mature processing would be characterized by a progressive, word-by-word increase in visual engagement with the target picture. The data support this hypothesis, but with important nuances. This contrast with reading development, where fixations decrease with age. This pattern likely reflects the higher cognitive demands of the sentence-picture matching paradigm, which requires constant reevaluation and comparison of competing images based on the analysis of subtle morphosyntactic features. Our data align with previous findings by Helo and colleagues, who showed that later phases of scene viewing lead to longer fixations, an effect that increases with age (Helo et al., 2014).

The less pronounced specificity in sentence processing, as assessed by oculomotor parameters for different grammatical types and processing stages in the younger age group (Figures 3, 4), may indicate an unspecific and immature strategy for analyzing incoming information, accompanied by lower response accuracy (Figure 2). This peculiarity in children’s oculomotor behavior may be linked to the incomplete development of the visual system, as full fixation control is not achieved until adolescence (Luna et al., 2008). With experience, the efficiency of search patterns improves, leading to greater concentration on the target image (Rebrejkina and Levkovich, 2024). Thus, selective and efficient eye-movement strategies that support real-time syntactic processing develop gradually with age.

Our interpretation of increased fixations on the target image as a reflection of active hypothesis testing and confirmation is supported by theoretical advancements in the visual world paradigm. Teruya (2019) demonstrated that looks to a referent are not an automatic reflection of lexical activation, but rather decisions made when activation exceeds a context-specific threshold (Teruya, 2019). This aligns with our conclusion that fewer fixations on the target image in younger children reflect a lack of sustained commitment to a hypothesis, rather than processing efficiency. Wendt and colleagues conceptualized the point at which participants consistently fixate on the target image as the “moment of decision”—the point of sentence understanding (Wendt et al., 2014). In our paradigm, older children and adults reach this decision point earlier (at the second word), allowing them to use subsequent words for verification, which is reflected in an increased number of saccades and longer fixations on the target image. This verification function of fixations aligns with real-world search studies showing that after the target template is activated, fixations serve to verify the match between the perceived object and the internal representation. Furthermore, Huo et al. (2023) showed that in picture-selection tasks based on auditorily presented sentence contexts, participants adopt a cautious strategy, waiting for unambiguous information before making a choice. This is a characteristic feature of our younger children, but is surpassed by the more active hypothesis testing seen in older participants.

This pattern of increased fixation time aligns with the verification function of eye movements. However, our linear mixed model (LMM) analysis, treating age as a continuous variable, revealed a more nuanced story when examining the total number of fixations (*all fix.*). While we observed a significant three-way interaction (Sentence Type × Word Position × Age) for this parameter (see Results 3.2.4), the *post hoc* analysis showed that the age-related increase in the number of fixations is not as uniform as the increase in duration.

Specifically, the number of fixations on the target image from the first to the last word significantly increased only for AR and PD sentences (Figure 4b). This finding is crucial: it suggests that the developmental refinement of visual strategies is not a global enhancement of all oculomotor metrics. Instead, the increase in the number of fixations is strategically deployed for the very sentence types that require a revision of the initial thematic-role hypothesis (AR and PD). Children are not just looking longer (fixation time); as they mature, they are also learning to sample visual information more frequently—to make more fixation “visits”—precisely when the syntactic structure demands reanalysis and comparison between the two potential referents. This interpretation is consistent with eye-tracking research showing that the number of fixations is particularly sensitive to task complexity (Huettig et al., 2011; Wendt et al., 2014; Teruya, 2019), and that such active comparison strategies are linked to the development of working memory and cognitive control (Hussey et al., 2017). The absence of a similar age effect for the number of fixations in simpler AD sentences suggests that once a structure is mastered, visual sampling becomes not only faster but also more economical, requiring fewer discrete fixations to confirm the initial hypothesis. This pattern of decreasing fixations with increasing expertise is well-documented in studies of expertise and visual search (Rebrejkina and Levkovich, 2024), supporting the view that the increase we observe in complex sentences is not a sign of inefficiency, but of active, hypothesis-driven engagement.

Our previous ERP study (Kruchinina et al., 2022a,b) compared AD and PD sentences and found significant differences between them only at the third word; the second word did not yield reliable effects. The present eye-tracking data extend this finding by showing that oculomotor indices of processing difficulty emerge as early as the second word, suggesting that eye movements are sensitive to earlier, more implicit stages of syntactic reanalysis that were not captured by ERPs in the smaller sample.

The shorter gaze fixations on target images observed in younger children likely indicate more superficial processing of target objects (Merzon et al., 2022). The limited executive and attentional capacities of younger children may constrain their use of incremental processing strategies. As children mature, developing executive control and syntactic competence enable them to execute more dynamic and purposeful eye movements, such as regressions and exploratory saccades, to verify and reanalyze hypotheses. The nuances of visual information processing are linked to the development of auditory-verbal working memory, cognitive flexibility, and cognitive inhibitory control (Hussey et al., 2017; Plotnikova et al., 2025). The transition to adult-like top-down behavioral control relies on the brain’s ability to efficiently integrate information, enabling the complex computations necessary for effective executive control of responses. Although executive functions begin to develop early, the consistent and flexible use of these systems continues to mature beyond adolescence (Lecce et al., 2024). Developmental constraints on visual fixation are linked to higher-order cognitive control processes, such as the ability to suppress eye movements in response to distracting stimuli (Luna et al., 2008). The age of nine marks a significant change in topological development and coincides with important cognitive, behavioral, and psychological milestones (Mousley et al., 2025). Crucially, these results demonstrate that while behavioral accuracy reaches adult levels by 8–10 years of age, the underlying oculomotor mechanisms—reflecting active hypothesis testing and verification—continue to develop. This dissociation makes eye tracking a uniquely sensitive tool for capturing the subtleties of maturing syntactic processing that are invisible to overt accuracy measures alone.

### 4.3 Sentence type and processing strategies

The results reveal distinct oculomotor patterns for different grammatical structures. Comprehension of canonical active direct (AD) sentences, which follow the preferred subject–verb–object (SVO) order, appears to be guided by this default hypothesis. Because the initial SVO interpretation is correct, these sentences require no reanalysis and are processed relatively effortlessly. For non-canonical structures, such as active reversed (AR) sentences (OVS), participants adopt a different strategy. Here, the case marker on the first noun provides an early morphosyntactic cue that identifies it as the object, allowing thematic roles to be assigned earlier and facilitating prediction (Cholewa et al., 2019). The most complex pattern emerged for passive direct (PD) sentences, where the initial noun in nominative case temporarily supports an SVO interpretation that is contradicted by the participle at the second word.

Our first hypothesis (H1) predicted that the second word would serve as a critical trigger for syntactic revision, with adults and older children showing increased oculomotor engagement on the target picture specifically at this position—particularly in sentences requiring thematic role reanalysis, such as PD and AR. The results provide strong support for this prediction, most clearly evident in the processing of PD sentences (e.g., “Zebra (NOM) pokrašena (participle) pandoj (INS)”; see Table 1). It is at the second word that the passive participle—a key morphosyntactic marker signaling the inversion of thematic roles—is encountered, forcing a revision of the initial SVO interpretation.

For adults and children aged 8–10 years, processing the second word in a PD sentence was accompanied by a significant decrease in fixation time, number of fixations, and number of saccades (Figure 3) compared to other sentence types. This pattern reflects rapid detection of the mismatch with the SVO hypothesis, immediate uptake of the morphosyntactic cue, and successful reanalysis of the visual scene to select the target picture. This aligns with previous findings that, in adults, sentence interpretation is already being formed during the second word (Kruchinina et al., 2024). The conflict with the default SVO hypothesis arises precisely at this position, triggering a more extensive verification process in which participants compare both pictures before committing to a choice. Consequently, PD sentences produce a distinct oculomotor signature overall: fewer saccades, fewer total fixations, shorter fixation durations, and more frequent returns to the target area of interest (AOI) compared to other sentence types. This is consistent with evidence that sentences requiring syntactic reanalysis systematically alter eye-movement patterns (Wendt et al., 2014).

Thus, H1 is confirmed: by age 8–10, children demonstrate sensitivity to the revision demands of the second word, although the efficiency of this revision, as indexed by the magnitude of oculomotor engagement, continues to mature into adulthood.

In contrast, 6–7-year-old children did not show this characteristic reanalysis signature at the second word. Their oculomotor differences in PD sentences were significantly less pronounced than in older children and adults, appearing only in fixation times and gaze returns (Figure 3). This pattern suggests that younger children detect the anomaly—the mismatch with the expected SVO structure—but their cognitive system is not yet capable of rapidly and correctly interpreting the passive participle and revising the initial hypothesis. As a result, they often fail to select the correct picture (Figure 2) or, on correct trials, appear to rely on a “good enough” interpretation (Ferreira and Patson, 2007) that supports accurate picture selection without being underpinned by full syntactic revision online. This interpretation is consistent with and extends our previous ERP studies, which showed that in 6-year-old children, the key to understanding the passive voice is the brain’s sensitivity to the inflections of the second noun (the third word in the sentence), reflected in differences in evoked potential amplitude between active (AD) and passive (PD) sentences (Kruchinina et al., 2022a).

The processing difficulties observed in 6–7-year-olds are open to multiple theoretical interpretations. From a generative perspective, they may reflect the computational demands of syntactic movement required in passive and object-initial constructions. From a usage-based perspective, the same pattern could arise from the lower frequency of these constructions in child-directed speech (Tomasello, 2003; Akhutina et al., 2017; Stankova et al., 2020). Our data cannot adjudicate between these accounts, as construction frequency was not experimentally manipulated. However, the cross-linguistic parallel with findings from Portuguese (Luegi et al., 2024)—where object-initial sentences also elicit online processing costs despite robust offline comprehension—suggests that the observed difficulties may reflect universal constraints on working memory and cue integration, rather than language-specific frequency effects alone. Future research manipulating both structural complexity and input frequency is needed to disentangle these possibilities.

Adults exhibited mature real-time sentence processing, characterized by dynamic modulation of oculomotor parameters from word to word and clear differentiation based on grammatical sentence type. Children aged 8–10, despite achieving adult-like behavioral accuracy, continued to show underlying oculomotor differences—particularly in fixation duration, fixation count, and regressions to the AOI (Figure 3)—indicating that the mechanisms supporting syntactic analysis are still maturing even after overt performance has reached adult levels. These residual differences suggest that the efficiency of reanalysis is constrained by the development of domain-general executive functions. The ability to rapidly suppress the initial SVO bias and reprogram the oculomotor response to verify the target picture relies on inhibitory control and cognitive flexibility, which follow a protracted developmental trajectory well into adolescence (Luna et al., 2008; Davidson et al., 2006).

In summary, the specificity of oculomotor behavior in relation to grammatical types develops gradually with age and is not yet fully mature by age 10. This underscores the prolonged development of the cognitive and linguistic systems that underpin syntactic processing, particularly for complex constructions like passives that require the revision of initial interpretive hypotheses.

## 5 Conclusion

This study demonstrates that the development of syntactic processing in Russian-speaking children undergoes a qualitative reorganization between the ages of 7 and 8 years. This reorganization is reflected in the transition to a more incremental, word-by-word processing strategy. While the first word in a sentence lacks sufficient information to determine thematic roles, the second word introduces critical morphosyntactic cues that allow for prediction, and the third word serves to confirm or revise these predictions. Selective and efficient eye-movement strategies that support this online syntactic processing develop gradually with age. For instance, the duration of fixations on the target picture increases from the beginning to the end of a sentence in 8–10-year-old children and adults, while the number of saccades increases only in adults. This pattern is not yet evident in 6–7-year-old children, who appear to require listening to the end of the sentence before making a decision.

Crucially, while behavioral accuracy for complex structures like passives reaches adult-like levels by age 8–10, eye-tracking reveals that the underlying online mechanisms continue to mature. The critical moment for this development is the processing of the second word in passive sentences, where morphosyntactic cues (the participle) force a revision of the initial canonical SVO hypothesis. Younger children (6–7 years) detect the anomaly but fail to revise it rapidly, whereas older children (8–10) and adults successfully integrate the cue, though adults do so with greater efficiency. Our findings, reinforced by linear mixed model analyses, show that the age-related increase in fixations and saccades is not a global phenomenon, but is specifically tied to mastering complex grammatical structures. This underscores that the transition to adult-like syntactic competence is a protracted process, shaped by both maturation and linguistic experience, and that oculomotor measures serve as sensitive, non-invasive markers of this developmental trajectory.

## 6 Limitations

This study has several limitations that should be considered when interpreting the findings.

First, the sample size, particularly in the child subgroups (*n* = 16 per age group), may have limited the statistical power for detecting higher-order interactions, despite the overall power exceeding 0.99 for the main analyses. Replication with larger samples is warranted.

Second, the cross-sectional design provides a snapshot of developmental differences but cannot capture within-individual change over time. Longitudinal studies are needed to trace the precise trajectory of syntactic maturation.

Third, our analysis focused exclusively on correct trials and on fixations directed to the target image. While this approach isolates successful processing, it does not account for the role of errors or for the distribution of attention between target and distractor. Future analyses could examine whether age-related differences in error patterns reflect distinct processing strategies.

Fourth, the study did not assess participants’ socioeconomic status, working memory capacity, or executive functions—factors known to influence both language development and oculomotor control (Hussey et al., 2017; Lecce et al., 2024). Including such measures in future work would help disentangle the cognitive mechanisms underlying the observed effects.

Fifth, the generalizability of our results is restricted by the inclusion of exclusively Russian-speaking participants. Russian is a morphologically rich language with flexible word order, where case marking plays a crucial role in thematic role assignment. Therefore, our findings may not directly extend to speakers of languages with different syntactic structures (e.g., fixed word order languages like English) or varying degrees of orthographic transparency.

Sixth, the eye-tracker’s sampling frequency (60 Hz) may have limited the temporal resolution of our oculomotor measures, potentially obscuring very rapid eye movements. Higher-speed tracking (e.g., 500–1,000 Hz) could provide finer-grained data, especially for saccade dynamics.

Finally, the use of three-word sentences and only four grammatical types restricts the generalizability of our findings to more complex syntactic constructions, such as relative clauses or sentences with inanimate arguments. Future studies should extend the paradigm to a wider range of linguistic structures.

## 7 Future directions

The present findings open several promising avenues for future research.

First, a natural extension of this work is to analyze gaze scan paths in greater detail. While we focused on aggregate measures such as fixation duration and saccade count, examining the sequential patterns of eye movements could reveal whether children and adults differ in how they allocate attention to the subject versus the object of an action. Such analyses would provide finer-grained insights into the maturation of thematic role assignment strategies and may uncover developmental changes in the order and timing of fixations to different regions of interest.

Second, given the cross-sectional nature of our study, longitudinal designs are essential to track the developmental trajectory of syntactic processing within the same individuals. Following children from age 6 through 10 would help pinpoint the timing of the transition to adult-like strategies and clarify whether this shift is gradual or marked by discrete stages.

Third, future studies should incorporate a broader range of syntactic constructions, such as relative clauses, sentences with inanimate arguments, or constructions involving syntactic ambiguity (e.g., participial phrases with multiple potential attachments). This would test the generality of our findings and reveal whether the processing difficulties observed for passive voice extend to other complex structures.

Fourth, including direct measures of working memory, inhibitory control, and cognitive flexibility would help elucidate the cognitive mechanisms underlying the observed oculomotor patterns. Recent evidence suggests that executive functions play a crucial role in online sentence processing (Hussey et al., 2017; Plotnikova et al., 2025), and individual differences in these abilities may account for the variability within age groups.

Fifth, multimodal approaches combining eye-tracking with neurophysiological methods (EEG, fNIRS, or fMRI) could provide a more comprehensive picture of syntactic development. While our previous ERP study (Kruchinina et al., 2022a,b) revealed neural sensitivity to passive voice at the third word, and the current eye-tracking data show earlier effects at the second word, simultaneous recordings could clarify the temporal relationship between attention allocation and neural computation.

Sixth, our finding that 6–7-year-olds fail to show target-directed oculomotor engagement at the critical verb position (word 2), even on correct trials, suggests that the absence of this word-by-word increase in fixation time could serve as a sensitive, implicit marker of delayed syntactic maturation. Future studies should explore whether this oculomotor signature can differentiate children with typical development from those at risk for Developmental Language Disorder, who often exhibit persistent difficulties with passive constructions.

Seventh, the adult group in our sample was predominantly female (31 out of 35 participants). While the child groups were more balanced (7 females in the 6–7-year-old group; 9 females in the 8–10-year-old group), the gender imbalance in the adult reference group should be acknowledged as a limitation. Although we are not aware of any evidence suggesting systematic gender differences in the oculomotor correlates of syntactic processing, we cannot entirely rule out the possibility that the observed adult patterns may be influenced by this imbalance. Future studies with more balanced adult samples are needed to confirm the generalizability of our findings.

Finally, cross-linguistic comparisons would be valuable to determine whether the developmental patterns observed in Russian—a morphologically rich language with flexible word order—generalize to languages with different syntactic structures. Such studies could distinguish universal aspects of syntactic maturation from language-specific constraints.

## Data Availability

The raw data supporting the conclusions of this article will be made available by the authors, without undue reservation.

## Footnotes

1
www.r-project.org
